# Deep neuromuscular blockade improves surgical conditions during low-pressure pneumoperitoneum laparoscopic donor nephrectomy

**DOI:** 10.1007/s00464-017-5670-2

**Published:** 2017-06-22

**Authors:** D. M. D. Özdemir-van Brunschot, A. E. Braat, M. F. P. van der Jagt, G. J. Scheffer, C. H. Martini, J. F. Langenhuijsen, R. E. Dam, V. A. Huurman, D. Lam, F. C. d’Ancona, A. Dahan, M. C. Warlé

**Affiliations:** 1 Division of Vascular and Transplant Surgery, Department of Surgery, Radboud University Medical Centre, Geert Grooteplein-Zuid 10, 6525 GA Nijmegen, The Netherlands; 2 Department of Surgery, Leiden University Medical Center, Albinusdreef 2, 2300 RC Leiden, The Netherlands; 3 Department of Anesthesiology, Radboud University Medical Centre, Geert Grooteplein-Zuid10, 6525 GA Nijmegen, The Netherlands; 4 Department of Anesthesiology, Leiden University Medical Centre, Albinusdreef 2, 2333 ZA Nijmegen, The Netherlands; 5 Department of Urology, Radboud University Medical Centre, Geert Grooteplein-Zuid 10, 6525 GA Nijmegen, The Netherlands

**Keywords:** Deep neuromuscular block, Laparoscopic donor nephrectomy, Laparoscopy, Low-pressure pneumoperitoneum, Pneumoperitoneum

## Abstract

**Background:**

Evidence indicates that low-pressure pneumoperitoneum (PNP) reduces postoperative pain and analgesic consumption. A lower insufflation pressure may hamper visibility and working space. The aim of the study is to investigate whether deep neuromuscular blockade (NMB) improves surgical conditions during low-pressure PNP.

**Methods:**

This study was a blinded randomized controlled multicenter trial. 34 kidney donors scheduled for laparoscopic donor nephrectomy randomly received low-pressure PNP (6 mmHg) with either deep (PTC 1–5) or moderate NMB (TOF 0–1). In case of insufficient surgical conditions, the insufflation pressure was increased stepwise. Surgical conditions were rated by the Leiden-Surgical Rating Scale (L-SRS) ranging from 1 (extremely poor) to 5 (optimal).

**Results:**

Mean surgical conditions were significantly better for patients allocated to a deep NMB (SRS 4.5 versus 4.0; *p* < 0.01). The final insufflation pressure was 7.7 mmHg in patients with deep NMB as compared to 9.1 mmHg with moderate NMB (*p* = 0.19). The cumulative opiate consumption during the first 48 h was significantly lower in patients receiving deep NMB, while postoperative pain scores were similar. In four patients allocated to a moderate NMB, a significant intraoperative complication occurred, and in two of these patients a conversion to an open procedure was required.

**Conclusions:**

Our data show that deep NMB facilitates the use of low-pressure PNP during laparoscopic donor nephrectomy by improving the quality of the surgical field. The relatively high incidence of intraoperative complications indicates that the use of low pressure with moderate NMB may compromise safety during LDN. Clinicaltrials.gov identifier: NCT 02602964.

## Introduction

Laparoscopic donor nephrectomy (LDN), in most countries the “gold standard” for live kidney donation, is associated with an improved quality of life, earlier return to work, and improved cosmetics [1–3]. Modifications of the standard transperitoneal laparoscopic approach, such as the hand-assisted and/or retroperitoneoscopic approach, have been introduced to refine the surgical technique. However, till date no evidence exists that the hand-assisted and/or retroperitoneoscopic approach improve the clinical outcome after LDN as compared to the standard transperitoneal procedure [4]. Less invasive technique modifications such as laparoendoscopic single site (LESS) or natural orifice transluminal (NOTES) improve the cosmetic result, but are associated with a longer learning curve and higher costs [5–8]. An alternative, simple, and therefore attractive method to refine the standard transperitoneal technique is the use of low-pressure PNP. There is accumulating evidence that low-pressure PNP reduces postoperative pain scores and analgesic consumption [9–12]. Furthermore, low-pressure PNP is better tolerated in cardiac-compromised patients. However, a lower intra-abdominal pressure during laparoscopy comes at a cost. It may compromise surgical conditions, such as working space and sight at the surgical field. A pilot study by our group showed that the use of low pressure during LDN was feasible, but increased duration of surgery [11]. A possible solution to this problem is the application of a deep neuromuscular block (NMB). Deep NMB may provide a better relaxation of the diaphragm and abdominal wall musculature as compared to a moderate NMB during laparoscopy and may thereby increase the space between the abdominal wall and intra-abdominal organs during laparoscopy. In a recent study by Kim et al., it was shown that the intra-abdominal pressure could be titrated from 12 mmHg to 9.3 mmHg in patients receiving a deep NMB during laparoscopic colorectal surgery, while intra-abdominal pressure was kept at 12 mmHg in patients allocated to a moderate NMB to maintain adequate surgical conditions [13]. Interestingly, this study showed that the surgical conditions were significantly better in patients receiving a deep NMB despite the use of a lower mean intra-abdominal pressure. However, Staehr-Rye et al. concluded that deep NMB only marginally improved surgical conditions during low-pressure laparoscopic cholecystectomy [14]. Therefore, the question whether deep NMB facilitates the use of low-pressure PNP during laparoscopy remains controversial. To address this issue, we perform a study in which we hypothesize that deep NMB improves surgical conditions during laparoscopic donor nephrectomy with low-pressure PNP.

## Methods

### Patients

This study was performed between April 2015 and February 2016 at the Leiden University Medical Center (Leiden, the Netherlands) and the Radboud University Medical Center (Nijmegen, the Netherlands). All adult patients eligible for LDN were approached at least 2 weeks before surgery. Exclusion criteria included insufficient knowledge of the Dutch language to read the patient information and fill out the questionnaires, chronic use of analgesics or psychotropic drugs, known or suspect allergy to rocuronium or sugammadex. The trial was registered at trials.gov (NCT 02602964).

### Ethics

Ethical approval for this study (Ethical Committee file number: 2014-1322/NL-number: 50874.091.14) was provided by the ‘Commissie Mensgebonden Onderzoek Arnhem-Nijmegen’, Geert Grooteplein-zuid 10, 6525 GA, Nijmegen, the Netherlands (Chairperson Prof. E. van Leeuwen) on November 10, 2014.

### Randomization and blinding

Patients were randomized using a computer-generated randomization code and assigned to either group 1: low-pressure PNP (6 mmHg) and deep NMB (PTC 1–5) or group 2: low-pressure PNP (6 mmHg) and normal NMB (TOF 0–1). Surgeons, scrub nurses, and the researchers were blinded for allocation of the treatment.

### Anesthesia

Anesthesia was induced by administering 1–3 mg/kg propofol and 0.2–0.5 µg/kg sufentanil. The TOF-watch (TOF-watch SX, MSD BV, the Netherlands) was calibrated before the administration of NMB agents. First, a tetanic ulnar nerve stimulation was applied, and subsequently the TOF-watch was calibrated. To ensure adequate calibration, 3 TOF measurements were performed; when these 3 measurements differed >5%, the TOF-watch was recalibrated. For patients in group 1, rocuronium 1 mg/kg and for patients in group 2, rocuronium 0.6 mg/kg was administered. 0.05–0.5 µg/kg/h Sufentanil and sevoflurane (1 MAC) were used to maintain anesthesia. For patients in group 1, a continuous infusion of rocuronium was used to maintain deep NMB. The infusion was started at 0.3 mg/kg/h but could be adjusted when post-tetanic count (PTC) was 0 or >5. PTC was measured every 15 min. In group 2, TOF was measured every 15 min, and in case of TOF >1, an extra dose of rocuronium was administered. In all patients, sugammadex 4 mg/kg was administered after surgery. Patients were extubated when TOF ratio was at least 90%.

### Surgical procedure

At the LUMC, a Veress needle was used to establish a pneumoperitoneum, and at Radboud University Medical Center, a Hasson trocar was introduced under direct vision. In both the hospitals, the camera trocar, two 5 mm trocars, and one 10/12 mm trocar were subsequently introduced. The hepatic or splenic flexure was mobilized. Gerota’s fascia was opened and the renal artery, vein, and ureter were identified. When present, the gonadal, suprarenal, and/or lumbal vein were clipped and transected. Subsequently, the kidney was mobilized and the ureter was transected. A pfannenstiel incision was made, the renal artery and vein were dissected using the endostapler^®^ or vascular clips. After extraction, the kidney was flushed at the back table using cold preservation solution. Afterward, the abdominal cavity was inspected and hemostasis was performed.

### Evaluation of perioperative conditions

After introduction of the camera trocar, after introduction of the third trocar, every 15 min during dissection of the kidney, and at transection of the renal artery, surgical conditions were evaluated using a modified Leiden-Surgical Rating Scale (L-SRS). The L-SRS is a Likert scale ranging from 1 to 5 where 1 indicates extremely poor conditions, 2 poor conditions, 3 acceptable conditions, 4, good conditions, and 5 optimal conditions [15]. The L-SRS aims to quantify the quality of the surgical field based on visibility, surgical space, muscle contractions, handling tactics, and patient movement. In two previous studies, the L-SRS was used in retroperitoneal surgery and consistent L-SRS scorings depending on the depth of NMB were observed without any effect of other factors such as duration of surgery, ventilator settings, and level of arterial PCO_2_ [15, 16]. Three separate scores were asked for (1) visibility, (2) working space, and (3) muscle contractions as well as an overall score. When the overall score was below 4 (good conditions), intra-abdominal pressure was increased in steps of 2 mmHg. For group 2, in case of insufficient perioperative conditions despite an intra-abdominal pressure of 12 mmHg, NMB could be converted to a deeper NMB. When insufficient peroperative surgical conditions persisted despite deep NMB and normal intra-abdominal pressure, the surgeon was allowed to handle according to regular protocols, e.g., further increase the intra-abdominal pressure and/or convert to open donor nephrectomy (ODN).

### Outcome measures

The primary outcome measure was the mean peroperative SRS, measured after trocar introduction and every 15 min thereafter. Secondary outcome measures included operation time (ORT), abdominal pressure, need to increase intra-abdominal pressure, first warm ischemia time (WIT1), estimated blood loss (EBL), perioperative complications, postoperative pain scores, postoperative complications, and postoperative serum creatinine levels. To assess whether the primary surgeon was adequately blinded, he was asked to guess at the end of the procedure whether deep or standard NMB was used.

### Sample size calculation and data analysis

An adapted version of the L-SRS was used for this study. A difference of 0.5 points on the SRS score was used as smallest clinical relevant difference. In the study by Martini et al., mean L-SRS was 4.0 points [15]. For sample size calculations, a standard deviation (SD) of 0.5 was used. A sample size of 17 patients per group was required to obtain a power of 80% with an alpha of 0.05. Continuous variables were expressed as mean ± SD; categorical data as number (percentage). Data were analyzed on an intention-to-treat basis. A Student *t* test was used to compare normally distributed data. All analyses were performed using SPSS version 22 (IMB Corp. Released 2013. IBM SPSS Statistics for Windows, Version 22.0. Armonk, NY: IBM Corp).

## Results

### Patient characteristics

A total of 34 patients were randomized. Patient demographics are shown in Table 1. There were no significant differences in baseline characteristics. A slight imbalance occurred during block randomization; therefore, 15 patients were randomized to deep NMB and 19 patients to standard NMB.

**Table 1:** Baseline characteristics

	Deep NMB (*n* = 15)	Standard NMB (*n* = 19)	*p* value
Male:female	9:6	13:6	0.61
BMI	24.7 ± 3.7	26.2 ± 3.7	0.22
Right kidney	5 (33%)	4 (21%)	0.42

### Per- and postoperative outcomes

**Fig. 1:**
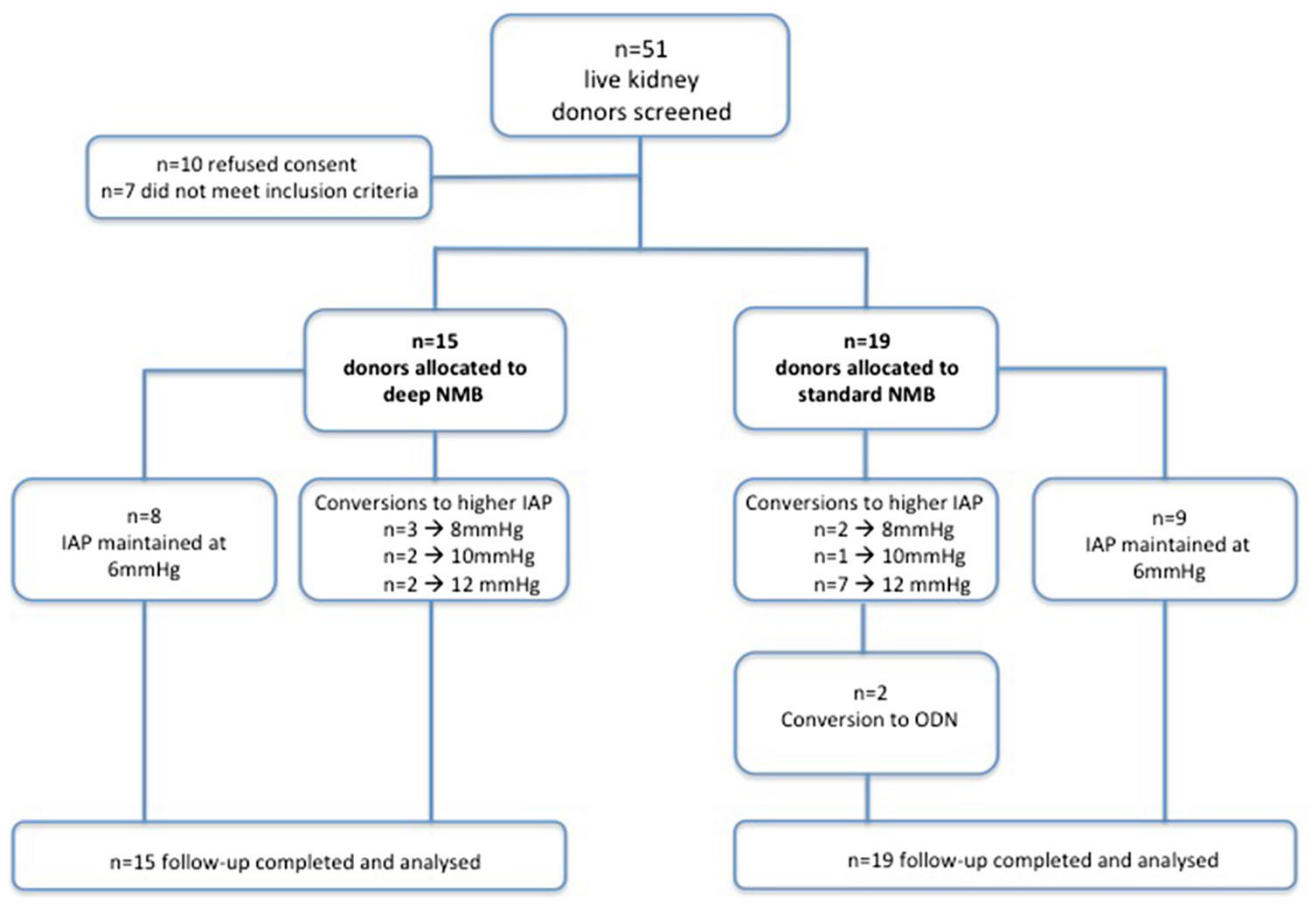
Study flow chart

Mean SRS was 4.5 in group 1 (deep NMB) versus 4.0 in group 2 (moderate NMB) (*p* = 0.01), Table 2. No significant differences were found in the following peroperative parameters: ORT, PNP duration, EBL, and WIT1. For the patients who received moderate NMB, 9 procedures were completed with low PNP at 6 mmHg, in ten procedures it was necessary to increase the pressure to 8 mmHg or higher (Fig. 1). Eight of 15 procedures with deep NMB were completed with low PNP at 6 mmHg. Mean abdominal pressure at the transection of the artery (final stage of the procedure) was 7.7 mmHg in the deep NMB group versus 9.1 mmHg in the standard NMB group (*p* = 0.21). Pain scores were comparable in both groups, see Table 3. However, opiate use on day 1 and cumulative opiate use after 48 h were significantly lower in the deep NMB group (*p* = 0.05).

**Table 2:** Peroperative parameters

	Deep NMB (*n* = 15)	Standard NMB (*n* = 19)	*p* value
Mean Surgical Rating Scale (0–5)			
SRS first trocar	4.3 ± 0.7	3.8 ± 0.8	0.07
SRS third trocar	4.3 ± 0.7	3.9 ± 0.8	0.13
SRS at 15 min	4.5 ± 0.5	4.1 ± 0.6	**0.03**
SRS at 30 min	4.4 ± 0.5	4.2 ± 0.5	0.12
SRS at 45 min	4.4 ± 0.5	4.2 ± 0.5	0.12
SRS at 60 min			
SRS at 75 min			
Overall SRS^1^	4.5 ± 0.5	4.0 ± 0.4	**0.01**
Mean Intra-abdominal pressure (mmHg)			
IAP first trocar	6.0	6.0	1.0
IAP third trocar	6.4 ± 1.1	7.2 ± 2.0	0.21
IAP at 15 min	6.5 ± 1.2	7.4 ± 2.1	0.18
IAP at 30 min	6.8 ± 1.5	7.7 ± 2.3	0.21
IAP at 45 min	6.9 ± 1.8	7.8 ± 2.5	0.26
IAP at 60 min			
IAP at 75 min			
Primary surgeon <50 LDNs	6	11	0.49
Operation duration (min)	143 ± 34.7	159 ± 45.4	0.26
PNP duration (min)	136 ± 63.9	138 ± 47.0	0.89
EBL (ml)	137 ± 199	331 ± 603	0.21
First WIT (s)	290 ± 118	372 ± 293	0.30

*p* values < 0.05 are given in bold

^a^Primary endpoint

**Table 3:** Postoperative pain and analgesics

	Deep NMB (*n* = 15)	Standard NMB (*n* = 19)	*p* value
Overall maximum pain score			
Postoperative 1 h	4.5 ± 2.5	4.8 ± 2.6	0.74
Postoperative day 1	4.5 ± 2.6	4.4 ± 2.4	0.91
Superficial wound component			
Postoperative 1 h	1.6 ± 1.4	1.2 ± 1.3	0.48
Postoperative 1 h (movement)	2.3 ± 1.6	2.8 ± 2.2	0.44
Postoperative day 1	1.0 ± 1.3	1.9 ± 1.8	0.13
Postoperative day 1 (movement)	2.3 ± 1.9	3.1 ± 2.4	0.27
Deep intra-abdominal component			
Postoperative 1 h	1.5 ± 1.7	1.1 ± 1.0	0.53
Postoperative 1 h (movement)	2.1 ± 1.9	1.8 ± 1.4	0.61
Postoperative day 1	1.2 ± 1.4	1.3 ± 1.4	0.79
Postoperative day 1 (movement)	1.9 ± 2.0	2.3 ± 1.8	0.60
Referred shoulder component			
Postoperative 1 h	0.0 ± 0.0	0.1 ± 0.3	0.20
Postoperative 1 h (movement)	0.1 ± 0.5	0.2 ± 0.4	0.87
Postoperative day 1	1.7 ± 2.5	1.9 ± 2.2	0.80
Postoperative day 1 (movement)	2.0 ± 2.7	2.1 ± 2.7	0.89
Analgesic medication			
Opiate use day 0 (mg)	17	19	0.55
Opiate use day 1 (mg)	4	9	**0.04**
Opiate use day 2 (mg)	1	3	0.19
Cumulative opiate use 48 h (mg)	22	31	**0.05**

*p* values < 0.05 are given in bold

### Safety

Conversion to ODN was necessary in two patients to control an arterial bleeding, Table 4. Both patients had been allocated to normal NMB. In the first patient, the pressure had been increased to 8 mmHg due to insufficient surgical conditions. Conversion to ODN was required due to iatrogenic injury of the renal upper pole artery. In the second patient, conversion was required after an iatrogenic injury of the renal artery. At this point, the intra-abdominal pressure was already increased to normal (12 mmHg) due to insufficient visibility. In both the cases, further postoperative recovery was uncomplicated. In two other patients allocated to a moderate NMB, other intraoperative complications occurred after conversion of the IAP to 12 mmHg for insufficient conditions. In one patient, a bleeding of a lumbal vein occurred and in another patient, the proximal ureter was pulled into the endostapler^®^ and transected during extraction of the kidney.

**Table 4:** Conversions, intra-, and postoperative complications

	Deep NMB (*n* = 15)	Standard NMB (*n* = 19)	*p* value
Conversions			
Conversions to higher IAP	7	10	1.0
8 mmHg	3	2	
10 mmHg	2	1	
12 mmHg	2	7	
Conversion to ODN	0	2	0.20
Intraoperative complications	0	4	0.11
Arterial bleeding^a^	0	2	
Venous bleeding	0	1	
Ureter transection	1	1	
Postoperative complications	0	3	0.61
Hematoma Pfannenstiehl	0	1	
Vasovagal collaps	0	1	
Transient neuralgia	1	0	
Hypertension	0	1	

^a^In both cases conversion to ODN

### Assessment of blinding

In 60% of the patients operated with deep NMB, the surgeon guessed that deep NMB was used. And when normal NMB was used, in 42% cases the correct depth of NMB was guessed.

## Discussion

This study shows that a deep NMB facilitates the use of low-pressure PNP during LDN by improving the quality of the surgical field. The surgical conditions as quantified by the Leiden-SRS were significantly better in patients allocated to a deep NMB. Similar results were found in another recent study by Kim et al. [14]. In the study by Kim et al., deep NMB was used in patients undergoing laparoscopic colorectal surgery with titration of the intra-abdominal pressure of 12 mmHg in patients with a moderate NMB, meaning that the insufflation pressure was kept at 12 mmHg in all patients allocated to a moderate NMB. In our study, the insufflation pressure was titrated from 6 mmHg until good or optimal surgical conditions were reached. In nine patients of 19 patients allocated to a moderate NMB, the procedure was completed with a pressure of 6 mmHg. This suggests that the titration of the insufflation pressure from low to high results in more procedures completed with a low insufflation pressure. However, in our study, we observed four intraoperative complications in patients allocated to a moderate NMB and in two cases a conversion to ODN was required due to severe bleeding. This strongly indicates that the safety of a low insufflation pressure with a moderate NMB is hampered.

A recent study by Madsen et al. showed that the incidence of referred shoulder pain was lower in women allocated to a low insufflation pressure and deep NMB (8 mmHg) as compared to women allocated to a standard insufflation pressure (12 mmHg) with a moderate NMB [17]. This is in line with a recent systematic review and meta-analysis revealing that the use of low-pressure PNP during laparoscopy is associated with reduced overall and referred shoulder pain scores after laparoscopy [10]. This study showed that patients allocated to a deep NMB consumed significantly less opiates during the first 48 h after surgery with similar pain scores. This reduction in opiate consumption could at least partly be attributed to the lower mean intra-abdominal pressure in patients allocated to a deep NMB (7.7 versus 9.1 mmHg). However, an alternative explanation might be that the use of a deep NMB directly affects postoperative pain scores [18]. In theory, a deep NMB allows an increased stretching of the abdominal wall muscle fibers during the insufflation of carbon dioxide [19]. Subsequently this may reduce stretch-induced abdominal pain after laparoscopy.

The main strengths of this study are related to its design as a randomized controlled study in which the blinding of the surgeons was accurate. Also a relatively homogenous population was studied, as live kidney donors are relatively young and in good health, thereby reducing the risk of confounding bias. An important limitation of our study is that the sample size is relatively small. Although the study is adequately powered regarding the primary endpoint, results regarding the secondary endpoints should be interpreted with care.

## Conclusion

In conclusion, our data show that deep NMB facilitates the use of low-pressure PNP during laparoscopic donor nephrectomy by improving the quality of the surgical field. Given the relatively high incidence of intraoperative complications and conversions to ODN, the safety of low-pressure PNP with moderate NMB may be hampered.

## Abbreviations

EBL: Estimated blood loss
HALDN: Hand-assisted laparoscopic donor nephrectomy
LDN: Laparoscopic donor nephrectomy
ORT: Operation time
NMB: Deep neuromuscular block
PNP: Pneumoperitoneum
PTC: Post-tetanic count
QoR40: Quality of recovery
TOF: Train-of-four
WIT1: First warm ischemia time
